# Minimally Invasive Epicardial Pacemaker Implantation in Neonates with Congenital Heart Block

**DOI:** 10.5935/abc.20170126

**Published:** 2017-10

**Authors:** Roberto Costa, Katia Regina da Silva, Martino Martinelli Filho, Roger Carrillo

**Affiliations:** 1 Instituto do Coração (InCor) do Hospital das Clínicas da Faculdade de Medicina da Universidade de São Paulo, São Paulo, SP - Brazil; 2 University of Miami - Miller School of Medicine, Miami - USA

**Keywords:** Heart Defects, Congenital, Infants, Newborns, Pacemaker, Artificial, Atrioventricular Block

## Abstract

**Background:**

Few studies have characterized the surgical outcomes following epicardial pacemaker implantation in neonates with congenital complete atrioventricular block (CCAVB).

**Objective:**

This study sought to assess the long-term outcomes of a minimally invasive epicardial approach using a subxiphoid access for pacemaker implantation in neonates.

**Methods:**

Between July 2002 and February 2015, 16 consecutive neonates underwent epicardial pacemaker implantation due to CCAVB. Among these, 12 (75.0%) had congenital heart defects associated with CCAVB. The patients had a mean age of 4.7 ± 5.3 days and nine (56.3%) were female. Bipolar steroid-eluting epicardial leads were implanted in all patients through a minimally invasive subxiphoid approach and fixed on the diaphragmatic ventricular surface. The pulse generator was placed in an epigastric submuscular position.

**Results:**

All procedures were successful, with no perioperative complications or early deaths. Mean operating time was 90.2 ± 16.8 minutes. None of the patients displayed pacing or sensing dysfunction, and all parameters remained stable throughout the follow-up period of 4.1 ± 3.9 years. Three children underwent pulse generator replacement due to normal battery depletion at 4.0, 7.2, and 9.0 years of age without the need of ventricular lead replacement. There were two deaths at 12 and 325 days after pacemaker implantation due to bleeding from thrombolytic use and progressive refractory heart failure, respectively.

**Conclusion:**

Epicardial pacemaker implantation through a subxiphoid approach in neonates with CCAVB is technically feasible and associated with excellent surgical outcomes and pacing lead longevity.

## Introduction

Permanent pacemaker implantation in neonates with congenital complete atrioventricular block (CCAVB) is technically challenging due to the small size of the patients, presence of concomitant structural heart defects, and rapid child growth. This results in a high complication rate, including lead fracture and pacing/sensing dysfunction.^1-7^ Fortunately, the number of children requiring pacemaker implantation in the first month of life is extremely low.^1-3^ This is one reason why the surgical outcomes in this subset of patients remain poorly elucidated.

Several age-specific factors may contribute to the occurrence of pacemaker-related complications in pediatric patients. First, pulse generators and leads are primarily designed for adults. Second, small vessel size and associated intracardiac defects make transvenous implantation difficult or impossible. Third, there is a significant disproportion between the size of the permanent device and the child's body size. Furthermore, the effects of growth on the leads and on the lead-myocardial junction result in a high incidence of exit block and lead fractures.^1-16^

Deciding on the best surgical approach for pacemaker implantation in neonates requires a thorough assessment and a highly experienced cardiac surgery team, as evidence-based guidelines are still unavailable.^1-9,15-20^ The purpose of this study was to assess the long-term outcomes of a minimally invasive epicardial approach using a subxiphoid access for pacemaker implantation in this patient population.

## Methods

### Patients

Between July 2002 and February 2015, a total of 16 consecutive neonates underwent epicardial pacemaker implantation in a cardiovascular referral center (Sao Paulo, Brazil). The Institutional Review Board of the institution approved this study. Device implantation was achieved through a minimally invasive subxiphoid incision.

Among the 16 patients included in the study, nine (56.3%) were female. Mean patient age was 4.7 ± 5.3 days (range, 1 to 23 days). Indications for cardiac pacing included signs of low cardiac output in four (25%), heart rate < 55 beats/minute in three (18.7%), and both conditions in nine (56.3%) patients. Patent foramen ovale or patent ductus arteriosus were detected in 12 (75.0%) infants, and atrial septal defects were detected in four (25.0%) of them. In four (25.0%) neonates, congenital heart defects were not detected before pacemaker implantation. One child had moderate-to-severe tricuspid regurgitation, and another had pulmonary stenosis. Baseline characteristics of these patients are summarized in Table 1.

**Table 1:** Baseline characteristics of neonates with congenital complete atrioventricular block who underwent epicardial pacemaker implantation

Pt	Sex	Fetal diagnosis	GA at birth	Birth weight (g)	Heart rate at birth (bpm)	Cardiac defect	Age (days) at PM implant	Maternal lupus/ autoantibodies +	PM indication
1	F	Y	36	2630	40	N	4	Y	Bradycardia
2	F	Y	38	3046	50	PFO, PDA	3	Y	Bradycardia, HF
3	F	Y	36	1950	48	PFO, PDA, PS	2	N	Bradycardia
4	M	Y	37	3895	50	N	2	Y	HF
5	M	Y	32	2680	42	N	1	Y	Bradycardia, HF
6	F	Y	37	2720	45	ASD, PDA	9	Y	Bradycardia, HF
7	M	N	38	2700	40	PFO, PDA	23	N	Bradycardia, HF
8	F	Y	38	2655	42	N	2	Y	Bradycardia, HF
9	M	Y	39	3200	50	PFO, PDA	3	N	Bradycardia, HF
10	F	Y	36	2780	56	PFO, PDA	1	Y	Bradycardia, HF
11	F	Y	37	2340	42	ASD, PDA	5	Y	HF
12	F	Y	38	3340	40	PFO, PDA	2	Y	Bradycardia, HF
13	M	Y	38	3060	70	PFO, PDA	4	Y	HF
14	M	Y	38	2360	64	PFO, ASD	4	N	HF
15	M	Y	39	3500	49	PFO, PDA	4	Y	Bradycardia
16	F	Y	37	2600	50	ASD, PDA	6	Y	Bradycardia, HF

ASD: atrial septal defect; bpm: beats per minute; F: female; g: grams; GA: gestational age (in weeks); HF: heart failure; M: male; N: no/absence; PDA: patent ductus arteriosus; PFO: patent foramen ovale; PM: pacemaker; PS: pulmonary stenosis; Pt: patient; Y: yes/presence.

CCAVB was diagnosed prenatally in 15 (93.8%) patients and after birth in one. Fetal echocardiography, performed in 15 (93.8%) cases, confirmed the diagnosis of CCAVB and also detected structural heart defects in two (12.5%) fetuses. Maternal dexamethasone or beta-sympathomimetic agents were administered in six (37.5%) cases due to signs of fetal myocardial dysfunction and/or fetal hydrops.

Eight infants were delivered preterm (32-37 weeks gestational age). Cesarean section was carried out in all cases, except the one case in which there was no previous diagnosis of CCAVB. Gestational age, weight, and heart rate at birth are described in Table 1.

Clinical diagnosis of autoimmune disease was present in 12 (75.0%) mothers (Table 1). Among them, eight (50.0%) tested positive for systemic lupus erythematosus and four (25.0%) had Sjögren’s syndrome. Neonatal lupus erythematosus was diagnosed in two infants. Increased levels of anti-Ro/SSA and anti-La/SSB antibodies were detected in 10 (62.5%) mothers, while in four (25.0%) mothers this test was not performed.

None of the neonates underwent temporary pacing. Two neonates had a pacemaker placed immediately after birth due to low cardiac output and severe bradycardia. The remaining cases were monitored closely in the neonatal intensive care unit. If the neonate had evidence of heart failure, low cardiac output, or heart rate < 55-beats/minute, infusion of dopamine was administered to postpone the time to pacemaker implantation.

### Surgical technique

All procedures were performed with patients under general anesthesia in the operating room. A 3 cm longitudinal incision was made at the insertion of the xiphoid process and advanced inferiorly toward the umbilicus. After resection of the xiphoid process, an inverted T-shaped pericardiotomy was performed.

A bipolar steroid-eluting epicardial lead (CapSure Epi 4968-35; Medtronic Inc., Minnesota, USA) was implanted in all neonates. Each of the two poles of the lead was affixed to the visceral epicardium with 5-0 polypropylene sutures. One of the poles was positioned on the diaphragmatic wall of the right ventricle. The other pole was implanted either on the anterior wall of the right ventricle or the inferior wall of the left ventricle.

**Figure 1:**
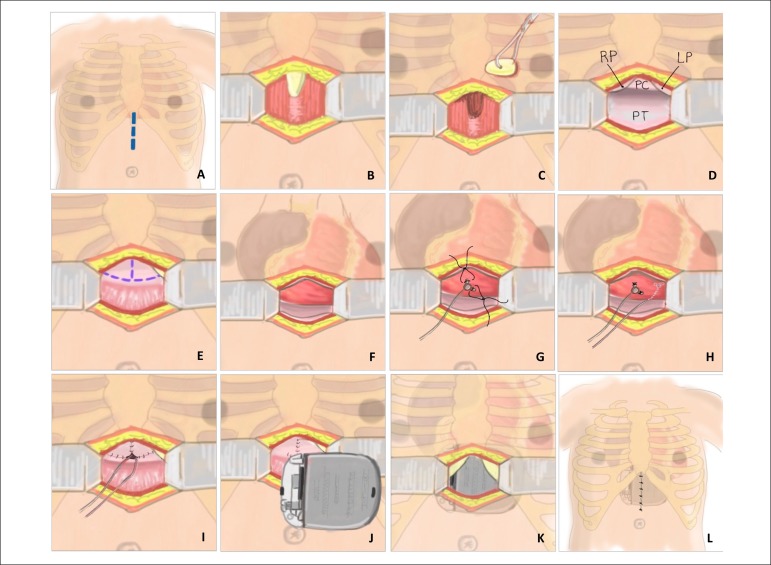
Epicardial pacemaker implantation in neonates through a subxiphoid approach. A: Midline incision in the skin, subcutaneous tissue, and aponeurosis of the rectus abdominis muscle; B: Xiphoid process view, which approximately occupies the upper half of the incision; C: Resection of the xiphoid process; D: Pericardial sac closed (PC), between the right pleura (RP), left pleura (LP) and the parietal peritoneum (PT); E: Inverted T-shaped pericardiotomy incision; F: Heart view after opening the pericardial sac and traction in the caudal direction; G: The bipolar steroid-eluting ventricular lead is directly affixed to the epicardium with two 5-0 polypropylene sutures; H: Position of the two poles of the lead: the cathode was positioned on the diaphragmatic wall of the right ventricle; the anode was implanted on the anterior wall of the right ventricle or on the inferior wall of the left ventricle; I: Pericardial sac already closed with the bipolar lead externalized in a rectilinear trajectory toward the epigastrium; J: Epigastric submuscular pulse generator pocket; K: Pulse generator positioned within the epigastric submuscular pocket and connected to the bipolar ventricular lead; L: Final aspect of the operation.

**Figure 2:**
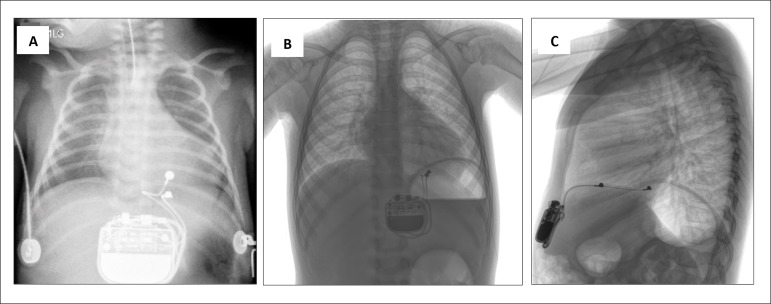
Chest radiographic projections displaying the radiologic appearance of epicardial pacemaker implantation immediately after the procedure (A) and 3 years later, in anteroposterior (B) and lateral projections (C).

Measurements of sensing, impedance, and capture threshold were obtained for both unipolar and bipolar configurations. Once satisfactory pacing and sensing parameters were achieved, the ventricular lead and the pulse generator (VVIR) were connected and placed within a pocket located in the submuscular region of the epigastrium (Figure 1). Lead excess was carefully accommodated under the pulse generator to leave its trajectory rectilinear to avoid excess in the pericardial sac or in the retrosternal space. The pulse generator was attached to the left rectus abdominis muscle. Pericardial drainage tubes were not used, and postoperative chest radiography confirmed proper lead location (Figure 2).

### Patients’ follow-up

All patients were followed up by a pediatric cardiology team and a cardiac pacing specialist. During follow-up, clinical assessment of all patients was performed, including careful evaluation of signs and symptoms related to heart failure. Patients with congenital heart defects were also evaluated regarding the optimal time for surgical repair.

Clinical follow-up and device interrogation visits were conducted every 6 months. In addition, subjects were periodically contacted by telephone and their medical records were regularly monitored.

Pacemaker programming was carried out according to individual patient clinical characteristics, and pacing energy was adjusted to allow for an optimal safety margin with respect to the ventricular pacing threshold. In the early follow-up period, the pacemaker was programmed at 110 to 120 beats per minute and this minimal heart rate was incrementally decreased in the chronic period, according to the individual characteristics and the childhood phase.

### Data collection and outcome variables

Study data were collected and managed using Research Electronic Data Capture (REDCap) software hosted in our institution’s server.^21,22^

The outcomes evaluated in the study included (1) intraoperative and immediate postoperative complications, or complications during the clinical follow-up period and (2) mortality from any cause. Quantitative variables are described as mean and standard deviation and qualitative variables as absolute and relative frequencies.

## Results

All procedures were successful with no perioperative lead dislodgment, bleeding, arrhythmias, or early deaths. Mean operating time was 90.2 ± 16.8 minutes (range, 65 to 120 minutes; median, 89 minutes). Four patients had hemodynamic instability, which was treated by decreasing the pacing rate and intravenous infusion of epinephrine (0.01 µg/kg/min).

The cathode of the ventricular lead was implanted on the inferior wall of the left ventricle and the diaphragmatic wall of the right ventricle in 10 (62.5%) and six (37.5%) neonates, respectively. The anode was implanted on the diaphragmatic or anterior wall of the right ventricle in 13 (81.3%) and three (18.8%) patients, respectively. One neonate underwent concomitant surgical closure of the patent ductus arteriosus by an independent incision (extra-pleural posterolateral thoracotomy). Excellent intraoperative pacing and sensing thresholds were obtained in all patients, as described in Table 2.

**Table 2:** Perioperative patient details

Pt	Total procedure time (minutes)	Pulse generator	Ventricular lead	Pacing site	R wave (mV)Uni/ Bi	Ventricular threshold at 0.5 ms (V)Uni/ Bi	Ventricular impedance (Ohms)Uni/ Bi	Endotracheal intubation(hours)	LOS in the ICU (days)
1	85	Altrua S601	4968-35	LV	12.5 / 12.5	0.5 / 0.5	695 / 896	28	16
2†	76	Microny	4968-35	LV	10.5 / 8.3	0.6 / 0.4	540 / 958	30	16
3	88	Microny	4968-35	LV	9.4 / 13.0	0.6 / 0.5	505 / 730	7	17
4	92	Microny	4968-35	RV	16.2 / 26.0	0.4 / 0.5	614 / 708	4	22
5	72	Microny	4968-35	RV	8.3 / 9.6	0.7 / 0.8	647 / 775	672	32
6	85	Microny	4968-35	LV	12.5 / 12.5	0.6 / 1.5	636 / 926	25	10
7	90	Microny	4968-35	LV	13.0 / 15.2	1.1 / 1.2	800 / 930	48	18
8	95	Microny	4968-35	RV	7.2 / 7.8	0.8 / 0.9	845 / 885	4	2
9	120	Microny	4968-35	RV	10.5 / 12.5	0.5 / 0.6	770 / 879	192	10
10	115	Microny	4968-35	LV	5.3 / 9.7	0.8 / 1.0	745 / 944	168	10
11	70	Microny	4968-35	LV	12.5 / 9.2	0.8 / 0.7	862 / 970	120	18
12	115	Microny	4968-35	RV	8.3 / 11.0	0.5 / 0.8	590 / 902	336	14
13	65	Microny	4968-35	LV	14.5 / 17.4	1.0 / 0.9	510 / 816	26	11
14	105	Microny	4968-35	LV	12.5/ 17.1	0.6 / 0.7	823 / 920	168	13
15	95	Microny	4968-35	RV	7.8 / 8.5	0.7 / 0.9	780 / 950	24	19
16	75	Microny	4968-35	LV	7.3 /9.6	0.6 /0.7	810 / 880	23	13

Bi: bipolar; LOS in the ICU: length of stay in the intensive care unit; LV: left ventricle; mV: millivolts; Pt: patient; RV: right ventricle; Uni: unipolar; V: volts

† Neonate underwent concomitant surgical closure of the patent ductus arteriosus.

A Microny II SR (St Jude Medical, California, USA) pulse generator was used in almost all patients. In only one case, an Altrua S601 SSIR (Boston Scientific, Minnesota, USA) pulse generator was chosen due to unavailability of the Microny device.

After pacemaker implantation, mechanical pulmonary ventilation was maintained for a minimum of 4 hours and a maximum of 30 days (mean, 117.2 ± 174.9 hours). One neonate was maintained on mechanical pulmonary ventilation for 30 days due to lung maturation problems. The length of stay in the neonatal intensive care unit ranged from 2 to 32 days (mean, 13.8 ± 7.0 days) and the total hospitalization length ranged from 7 to 49 days (mean, 23.4 ± 12.0 days).

Minimal superficial wound infection was the only procedure-related complication observed in our patients, occurring in three (18.8%) neonates. Other complications observed included pulmonary infection in two (12.5%), atelectasis in one (6.3%), urinary tract infection in one (6.3%), and renal failure in one (6.3%) neonate who also had superior vena cava thrombus treated with thrombolysis.

The patients were followed individually for 4.1 ± 3.9 years (range, 12 days - 12.7 years, median, 3.7 years). There were two deaths. One occurred 12 days after pacemaker implantation due to bleeding complications secondary to thrombolytic use. The other patient, who was being followed in another hospital, died of progressive refractory heart failure 325 days postoperatively.

Overall, 11 children remain without signs/symptoms of heart failure or need for cardiovascular medication. Two children underwent surgical repair of congenital heart defects. Percutaneous pulmonic valvuloplasty was performed in a 2-month-old girl with pulmonary valve stenosis. This resulted in rupture of a tricuspid valve papillary muscle and required urgent surgical repair of the pulmonic and tricuspid valve. A 4-year-old girl underwent surgical mitral valve repair and closure of an atrial septal defect. Concomitantly, this child was upgraded from a single-chamber to a dual-chamber pacemaker by using the previous ventricular lead and the same epigastric pulse generator pocket. Finally, a 5-year-old boy presented with refractory heart failure and was upgraded from a single-chamber device to a biventricular device for cardiac resynchronization therapy and 7 months later underwent heart transplantation (Table 3).

**Table 3:** Long-term outcomes after epicardial pacemaker implantation in neonates with congenital heart block

Pt	Follow-up time (years)	Surgical complications	Clinical complications	Medication use	NYHAFC	Generator replacement	Upgrade	LVEF	Surgical repair of intracardiac defect
1	4.2	N	N	N	I	N	N	0.51	N
2	1.1	N	N	N	I	N	N	0.67	N
3	0.8	N	N	Furosemide, spironolactone	I	N	N	0.61	Y
4	10.7	N	N	N	I	Y (7.2 years after PM implant)	N	0.66	N
5	5.0	N	N	N	I	N	N	0.71	N
6	4.2	Superficial wound infection	N	Furosemide, spironolactone	I	Y (4.0 years after PM implant)	DDD (4.0 years after PM implant)	0.67	Y
7	2.5	N	N	N	I	N	N	0.66	N
8	12.7	Superficial wound infection	N	N	I	Y (9.0 years after PM implant)	N	0.74	N
9	5.9	N	Heart transplant (5.9 years after PM implant)	Furosemide, spironolactone, carvedilol, captopril	III	Y (5.2 years after PM implant)	CRT-P(5.2 years after PM implant)	0.33	N
10	10.2	Superficial wound infection	N	N	I	Y (3.9 years after PM implant)	N	0.71	N
11	4.0	N	N	N	I	N	N	0.64	N
12	-	N	Death(12 days after PM implant)	Furosemide, amiodarone	IV	N	N	-	N
13	3.5	N	N	N	I	N	N	0.75	N
14	0.9	N	Death (325 days after PM implant)	N	I	N	N	0.65	N
15	0.4	N	N	Furosemide	I	N	N	0.75	N
16	0.8	N	N	N	I	N	N	0.68	N

CRT-P: cardiac resynchronization therapy; DDD: dual-chamber pacemaker; LVEF: left ventricular ejection fraction; N: no; NYHA FC: New York Heart Association Functional Class; PM: pacemaker; Pt: patient; Y: yes.

During follow-up, none of the subjects experienced loss of capture, lead dislodgement, or lead fracture. None of the patients displayed pacing or sensing dysfunction, and all pacemaker parameters remained stable throughout the follow-up period. Three children underwent pulse generator replacement due to normal battery depletion at 4.0, 7.2, and 9.0 years of age without the need for ventricular lead replacement (Table 3).

An echocardiogram confirmed normal cardiac anatomy and normal left ventricular function in five (31.3%) children. Among the cases with intracardiac defects, only two underwent surgical repair due to hemodynamic compromise. Of the 13 (81.3%) patients who remain in follow-up, only one was found to have reduced left ventricular ejection fraction (LVEF = 0.51).

**Figure 3:**
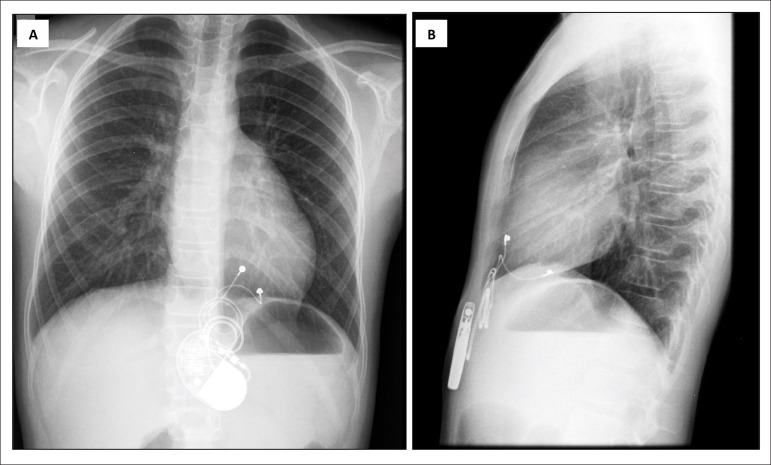
Chest radiographic projections displaying the appearance of epicardial pacemaker 10 years later, in anteroposterior (A) and lateral projections (B).

At the last follow-up, the electrocardiogram confirmed sinus rhythm with atrioventricular dissociation in all patients. Chest radiography revealed proper device location, lead integrity, and cardiac silhouettes within normal limits (Figure 3).

## Discussion

The use of cardiac pacing in neonates is still an area of significant controversy. Opinions differ with respect to the ideal pacing mode, the best surgical approach to pace the heart of small infants, the optimal lead choice which provides the best short and long-term outcomes, and the appropriate strategy to accommodate the pulse generator in this subset of patients.^1-9,15-20^

Traditionally, an epicardial approach has been preferred, though access options (sternotomy, lateral thoracotomy, subxiphoid) may vary.^1-18^ On the other hand, the feasibility of transvenous pacemaker implantation has been described in neonates, either by the tributaries of the superior vena cava or via the branches of the iliac veins.^3,10,13,14,20^ The disproportion between the small body size and the device dimensions prevents placement of the pulse generator in the chest wall. Therefore, to prevent pocket-related problems in neonates, pulse generators are usually placed in the abdominal wall.^12-18^

The debate regarding the optimal pacing mode for neonates is still ongoing. In most experts' opinion, a single-chamber ventricular system is the first choice, reserving dual-chamber systems or even cardiac resynchronization therapy for children with impaired left ventricular function or poor adjustment to single-site ventricular pacing.^18-20^ To date, few studies have recommended the use of more sophisticated pacing modes or cardiac resynchronization therapy as an initial strategy.^4,9,19^

Regardless of the surgical approach and pacing mode, device-related complications are common during follow-up. Although pocket-related complications, in particular, erosions or thinning of the skin are more frequent when the device is implanted in the chest wall, abdominal pockets may also be associated with complications.^15,17,18^

It is worth highlighting that lead fracture remains an important determinant of lead survival and is directly associated with the patient's growth.^3,4,7,10-16,20^ Overall, standard epicardial penetrating leads have been associated with a high incidence of increased pacing thresholds following implantation, requiring early lead or pulse generator replacement. Recent studies have shown that steroid-eluting leads are associated with a lower rate of lead failure.^11,12^

The technique described in this article aims to increase the safety of pacemaker use in neonates in four main ways: (1) reduction in surgical trauma by not opening the sternum or intercostal spaces; (2) safe approach and good cosmetic result for pulse generator accommodation in the preperitoneal space submuscularly; (3) reduction in fibrosis at the lead-myocardial junction by the use of steroid-eluting leads; (4) reduction in the effect of the child's growth on the leads and on the lead-myocardial junction by using a rectilinear trajectory and by ensuring proximity between the lead and pulse generator.

In our study, all operations were successful, and there were no perioperative complications. In addition, there were no complications related to surgical technique during the follow-up period (maximum of 12 years). In particular, there were no pocket-related complications (infection or skin erosion); lead-related complications (lead fracture), increases in pacing thresholds, or early battery depletion. Finally, measurements of sensing, pacing, and impedance remained satisfactory during the follow-up period.

Despite the use of single-site ventricular pacing, clinical signs of heart failure or echocardiographic abnormalities were not observed at last follow-up evaluation in 13 of the 16 neonates included in this study. In cases where hemodynamic compromise secondary to intracardiac defects was detected, surgical repair completely reversed this condition. Two patients developed severe ventricular dysfunction; one underwent a heart transplant and another died.

Within our study, there are several limitations. The main one is the small number of cases, inherent to the rarity of CCAVB and other causes of bradyarrhythmias requiring pacemaker implantation during the neonatal period. Second, the lack of a gold-standard surgical technique for pacemaker implantation in neonates does not allow for the formation of a control group with which to compare results. Even in larger centers, it is nearly impossible to conduct a study to compare outcomes between different techniques of pacemaker implantation in this subset of patients. Finally, all procedures were performed by the same surgeon. This lack of operator variability may have influenced surgical results.

## Conclusion

Epicardial pacemaker implantation through a subxiphoid approach in neonates with CCAVB is technically feasible and results in excellent surgical outcomes and pacing lead longevity. In addition, this surgical approach solves two of the main challenges related to permanent cardiac pacing in neonates: pocket and lead-related complications.

## References

[1] McLeod KA (2010). Cardiac pacing in infants and children. Heart.

[2] Takeuchi D, Tomizawa Y (2013). Pacing device therapy in infants and children a
review. J Artif Organs.

[3] Villain E, Martelli H, Bonnet D, Iserin L, Butera G, Kachaner J (2000). Characteristics and results of epicardial pacing in neonates and
infants. Pacing Clin Electrophysiol.

[4] Silvetti MS, Di Carlo D, Ammirati A, Placidi S, Di Mambro C, Ravà L (2015). Left ventricular pacing in neonates and infants with isolated
congenital complete or advanced atrioventricular block short- and
medium-term outcome. Europace.

[5] Shepard CW, Kochilas L, Vinocur JM, Bryant R, Harvey BA, Bradley S (2012). Surgical placement of permanent epicardial pacing systems in very
low-birth weight premature neonates a review of data from the pediatric
cardiac care consortium (PCCC). World J Pediatr Congenit Heart Surg.

[6] Glatz AC, Gaynor JW, Rhodes LA, Rychik J, Tanel RE, Vetter VL (2008). Outcome of high-risk neonates with congenital complete heart
block paced in the first 24 hours after birth. J Thorac Cardiovasc Surg.

[7] Aellig NC, Balmer C, Dodge-Khatami A, Rahn M, Prêtre R, Bauersfeld U (2007). Long-term follow-up after pacemaker implantation in neonates and
infants. Ann Thorac Surg.

[8] Dodge-Khatami A, Kadner A, Dave H, Rahn M, Prêtre R, Bauersfeld U (2005). Left heart atrial and ventricular epicardial pacing through a
left lateral thoracotomy in children a safe approach with excellent
functional and cosmetic results. Eur J Cardiothorac Surg.

[9] Kelle AM, Backer CL, Tsao S, Stewart RD, Franklin WH, Deal BJ (2007). Dual-chamber epicardial pacing in neonates with congenital heart
block. J Thorac Cardiovasc Surg.

[10] Sachweh JS, Vazquez-Jimenez JF, Schöndube FA, Daebritz SH, Dörge H, Mühler EG (2000). Twenty years' experience with pediatric pacing: epicardial and
transvenous stimulation. Eur J Cardiothorac Surg.

[11] Silvetti MS, Drago F, De Santis A, Grutter G, Ravà L, Monti L (2007). Single-centre experience on endocardial and epicardial pacemaker
system function in neonates and infants. Europace.

[12] Udink ten Cate F, Breur J, Boramanand N, Crosson J, Friedman A, Brenner J (2002). Endocardial and epicardial steroid lead pacing in the neonatal
and paediatric age group. Heart.

[13] Murayama H, Maeda M, Sakurai H, Usui A, Ueda Y (2008). Predictors affecting durability of epicardial pacemaker leads in
pediatric patients. J Thorac Cardiovasc Surg.

[14] Welisch E, Cherlet E, Crespo-Martinez E, Hansky B (2010). A single institution experience with pacemaker implantation in a
pediatric population over 25 years. Pacing Clin Electrophysiol.

[15] Lichtenstein BJ, Bichell DP, Connolly DM, Lamberti JJ, Shepard SM, Seslar SP (2010). Surgical approaches to epicardial pacemaker placement does pocket
location affect lead survival?. Pediatr Cardiol.

[16] Janousek J, Kubus P (2014). What's new in cardiac pacing in children. Curr Opin Cardiol.

[17] Costa R, Filho MM, Tamaki WT, Crevelari ES, Nishioka SD, Moreira LF (2003). Transfemoral pediatric permanent pacing: long-term
results. Pacing Clin Electrophysiol.

[18] Motonaga KS, Dubin AM (2014). Cardiac resynchronization therapy for pediatric patients with
heart failure and congenital heart disease a reappraisal of
results. Circulation.

[19] Janousek J, van Geldorp IE, Krupicková S, Rosenthal E, Nugent K, Tomaske M (2013). Working Group for Cardiac Dysrhythmias and Electrophysiology of
the Association for European Pediatric Cardiology Permanent cardiac pacing
in children: choosing the optimal pacing site: a multicenter study. Working
Group for Cardiac Dysrhythmias and Electrophysiology of the Association for
European Pediatric Cardiology. Circulation.

[20] Silvetti MS, Drago F, Rava L (2010). Determinants of early dilated cardiomyopathy in neonates with
congenital complete atrioventricular block. Europace.

[21] Harris PA, Taylor R, Thielke R, Payne J, Gonzalez N, Conde JG (2009). Research electronic data capture (REDCap) - A metadata-driven
methodology and workflow process for providing translational research
informatics support. J Biomed Inform.

[22] Silva KR, Costa R, Crevelari ES, Lacerda MS, Albertini CMM, Martinelli Filho M (2013). Glocal clinical registries pacemaker registry design and
implementation for global and local integration - methodology and case
study. PLoS One.

